# Prominent microglial inclusions in transgenic mouse models of α-synucleinopathy that are distinct from neuronal lesions

**DOI:** 10.1186/s40478-020-00993-8

**Published:** 2020-08-12

**Authors:** Gaye Tanriöver, Mehtap Bacioglu, Manuel Schweighauser, Jasmin Mahler, Bettina M. Wegenast-Braun, Angelos Skodras, Ulrike Obermüller, Melanie Barth, Deborah Kronenberg-Versteeg, K. Peter R. Nilsson, Derya R. Shimshek, Philipp J. Kahle, Yvonne S. Eisele, Mathias Jucker

**Affiliations:** 1 German Center for Neurodegenerative Diseases (DZNE), Tübingen, Germany; 2 Department of Cellular Neurology, Hertie Institute for Clinical Brain Research, University of Tübingen, Tübingen, Germany; 3 Graduate School of Cellular and Molecular Neuroscience, University of Tübingen, Tübingen, Germany; 4 Department of Physics, Chemistry and Biology IFM, Linköping University, Linköping, Sweden; 5 Novartis Institutes for BioMedical Research, Novartis Pharma AG, Basel, Switzerland; 6 Department of Neurodegeneration, Hertie Institute for Clinical Brain Research, University of Tübingen, Tübingen, Germany; 7 Department of Medicine, University of Pittsburgh, Pittsburgh, PA USA

**Keywords:** Synuclein, Microglia, Inclusion, Prion-like, Amyloid, Conformation, Parkinson’s disease

## Abstract

Alpha-synucleinopathies are a group of progressive neurodegenerative disorders, characterized by intracellular deposits of aggregated α-synuclein (αS). The clinical heterogeneity of these diseases is thought to be attributed to conformers (or strains) of αS but the contribution of inclusions in various cell types is unclear. The aim of the present work was to study αS conformers among different transgenic (TG) mouse models of α-synucleinopathies. To this end, four different TG mouse models were studied (Prnp-h[A53T]αS; Thy1-h[A53T]αS; Thy1-h[A30P]αS; Thy1-mαS) that overexpress human or murine αS and differed in their age-of-symptom onset and subsequent disease progression. Postmortem analysis of end-stage brains revealed robust neuronal αS pathology as evidenced by accumulation of αS serine 129 (p-αS) phosphorylation in the brainstem of all four TG mouse lines. Overall appearance of the pathology was similar and only modest differences were observed among additionally affected brain regions. To study αS conformers in these mice, we used pentameric formyl thiophene acetic acid (pFTAA), a fluorescent dye with amyloid conformation-dependent spectral properties. Unexpectedly, besides the neuronal αS pathology, we also found abundant pFTAA-positive inclusions in microglia of all four TG mouse lines. These microglial inclusions were also positive for Thioflavin S and showed immunoreactivity with antibodies recognizing the N-terminus of αS, but were largely p-αS-negative. In all four lines, spectral pFTAA analysis revealed conformational differences between microglia and neuronal inclusions but not among the different mouse models. Concomitant with neuronal lesions, microglial inclusions were already present at presymptomatic stages and could also be induced by seeded αS aggregation. Although nature and significance of microglial inclusions for human α-synucleinopathies remain to be clarified, the previously overlooked abundance of microglial inclusions in TG mouse models of α-synucleinopathy bears importance for mechanistic and preclinical-translational studies.

## Introduction

Accumulation of α-synuclein (αS) aggregates is a pathological hallmark of a group of neurodegenerative diseases called α-synucleinopathies. αS is the major component of Lewy bodies and Lewy neurites, which are intracellular inclusions found in neurons of patients with Parkinson’s disease (PD) and dementia with Lewy bodies (DLB). Apart from the neuronal Lewy pathology, filamentous αS also accumulates in oligodendrocytes to form glial cytoplasmic inclusions (GCIs or Papp-Lantos bodies) found primarily in multiple system atrophy (MSA) [1]. Furthermore, αS-positive cytoplasmic aggregates have been reported in astroglial cells of PD and DLB as well as MSA [2]. Indirect evidence has suggested that the diverse nature of α-synucleinopathies may be characterized by distinct conformers (or strains) of αS aggregates [3–9]. What is more, structural analysis has revealed the presence of different filament structures of αS aggregates derived from MSA and DLB brains [10]. A majority of aggregated αS is phosphorylated at serine 129 (p-αS) [11], therefore antibodies directed against p-αS are commonly used as a surrogate marker of αS pathology.

αS is a 140 amino acid protein and is primarily expressed in neurons where it is enriched at the presynaptic terminal [1, 12]. Several missense mutations in the *SNCA* gene encoding αS have been linked to rare familial forms of PD and DLB. The amino acid substitutions alanine-to-threonine at codon 53 (A53T) and alanine-to-proline at codon 30 (A30P) both give rise to early-onset PD [13, 14]. Morphological differences between A53T- and A30P-mutated αS fibrils have been demonstrated in vitro [15–18], although their relevance for human disease pathogenesis remains uncertain. Subsequently, numerous transgenic (TG) mouse models overexpressing human A53T or A30P αS under various promoters have been generated that develop neuronal Lewy-like pathology and motor symptoms that resemble PD [19–21].

Here, we compare disease progression, as well as cellular and structural features of αS lesions in four TG lines: Prnp-h[A53T]αS (in the literature also referred to as ‘M83’) [22], Thy1-h[A53T]αS [23], Thy1-h[A30P]αS [24, 25], and Thy1-mαS TG mice [26]. While disease onset and progression differed among the TG lines, morphological appearance and regional distribution of αS lesions did not reveal robust differences. Intriguingly, however, in addition to the neuronal αS lesions, we found abundant αS-immunoreactive inclusions in microglia and this in all four TG mouse lines. Microglial inclusions differed from neuronal inclusions in morphological and conformational features.

## Materials & methods

### Mice

The following TG mouse lines were used: Prnp-h[A53T]αS [22], Thy1-h[A53T]αS [23], Thy1-h[A30P]αS [24], and Thy1-mαS [26]. The Prnp-h[A53T]αS line expresses human (h) αS with the A53T mutation under the control of the mouse prion protein promoter (Prnp) generated on the C57BL/6 x C3H background. Hemizygous Prnp-h[A53T]αS mice were purchased from The Jackson Laboratory (Bar Harbor, ME, USA) and bred to generate homozygous offspring for the study. The Thy1-h[A30P]αS line expresses human αS with the A30P mutation under the control of the neuron-specific murine Thy-1 promoter generated on the C57BL/6 J background. These mice are routinely maintained in our mouse facility and homozygous mice were produced by breeding homozygous pairs. The Thy1-h[A53T]αS line expresses the human αS transgene harboring the A53T mutation under the control of the murine Thy-1 promoter and the Thy1-mαS line is transgenic for an overexpression of the mouse (m) wildtype αS driven by the murine Thy-1 promoter, each of those lines was generated on the C57BL/6 J background. Both lines were obtained from Novartis (Basel) and transferred to our facility. All Thy1-h[A53T]αS and Thy1-mαS mice used in the studies were hemizygous and produced by breeding hemizygous males with C57BL/6 J females. Care was taken that both male and female mice were used at an equal proportion for all the experiments but their use was subjected to availability. All mice were kept under specific pathogen-free conditions and maintained on a 12 h light/dark cycle with food and water ad libitum. The experimental procedures were undertaken in compliance with the veterinary office regulations of Baden-Württemberg (Germany) and approved by the local Animal Care and Use Committees.

### Determination of symptom onset, disease duration, and humane endpoint

A score sheet with a grading scale was used to evaluate and record the occurrence of motor signs in these mice. Rapid changes in body weight were used as clinical parameters to define the humane endpoints (i.e. loss of > 20% of the initial weight). For that purpose, mice were weighed weekly and checked for the onset of motor symptoms by using established criteria for neurodegenerative phenotypes in mice [27]. The behavioral assessment of mice was done first in open cages where the general activity and movements were observed. Mice were then placed on a grid to check for motor impairment and to assess putative signs of ill health. The symptomatic phase typically comprised several stages of severity. Initially, the mice showed a disturbance in balance and gait, culminating in ataxia. As the movements became slower, tremor and rigidity were often seen. At the end-stage of the illness, partial paralysis of hind limbs occurred, at which the mice were sacrificed. With the appearance of the first symptoms, mice were provided with wet food pellets in the cage. Disease duration was determined as the days between the occurrence of the first symptoms and above defined the-end-stage of the illness.

### Tissue processing

Brains were removed after the animals were deeply anesthetized and transcardially perfused with ice-cold PBS (0.1 M). For immunohistochemistry, one brain hemisphere was immersion-fixed for 48 h in 4% paraformaldehyde with PBS, then cryoprotected in 30% sucrose in PBS for an additional 2 days. After freezing, 25 μm-thick sagittal sections were serially cut through the entire hemisphere using a freezing-sliding microtome (Leica Microsystems). The sections were stored at − 20 °C in cryoprotection solution (35% ethylene glycol, 25% glycerol in PBS). For biochemical analysis, the other hemisphere was immediately snap frozen on dry ice and stored at − 80 °C.

### Brain extracts

Extracts were prepared as described previously [28]. The A30P extract was derived from spontaneously ill Thy1-h[A30P]αS females (16–20 months). After removal of the forebrain and cerebellum, the brainstem was immediately fresh-frozen on dry ice and stored at − 80 °C until use. Tissue was then homogenized (Precellys®24, Bertin Technologies, France) at 10% (w/v) in sterile, phosphate-buffered saline (PBS, Lonza, Switzerland), vortexed and centrifuged at 3000 *x* g for 5 min. The supernatant was aliquoted and immediately frozen. For all following experiments, the 10% (w/v) extract was used. The wildtype extract was derived from aged C57BL/6 J mice (24–26 months old).

### Stereotactic injection of brain extracts

αS host mice were anaesthetized with a mixture of ketamine (100 mg/kg body weight) and xylazine (10 mg/kg body weight) in saline and administered carprofen (5 mg/kg body weight) prior to surgery. Stereotactic injections were performed manually with a Hamilton syringe bilateral (2.5 μl of brain extract per side) into the hippocampus / dentate gyrus (AP − 2.5 mm, ML ±2.0 mm, DV − 1.8 mm) of Thy1-h[A30P]αS mice. Injection speed was 1.25 μl/minute. The needle was kept in place for an additional 2 min before it was slowly withdrawn. The surgical area was cleaned with sterile saline, the incision was sutured, and the mice were monitored until recovery from anesthesia. Injections were performed at the age between 2 and 4 months.

### Histology and immunohistochemistry

In preparation of immunolabeling, the brain sections were washed with Tris-buffered saline (TBS, 0.1 M, pH 7.4) and mounted onto microscopic glass slides (SuperFrost Plus, Langenbrinck, Germany). After treating the sections with 3% H_2_O_2_ (Applichem, Darmstadt, Germany) in TBS for 30 min to block the endogenous peroxidase, antigenicity was enhanced by boiling the sections in 10 mM citrate buffer (1.8 mM citric acid, 8.2 mM trisodium citrate, pH 6.0) at 90 °C for 35 min. Unspecific binding sites were blocked by using 5% normal goat serum in 0.3% Triton-X100 (Sigma-Aldrich, Steinheim, Germany) in TBS for 30 min at RT. To detect accumulating αS phosphorylated at serine 129 (p-αS), primary antibody rabbit monoclonal anti-p-αS (EP1536Y, Epitomics, Burlingame, CA, USA) was used at 1:750 dilution and incubated overnight at 4 °C. The following day, biotinylated secondary antibody (goat anti-rabbit biotinylated IgG, Vector laboratories, Burlingame, CA, USA) was added at 1:400 onto the sections and incubated for 45 min at RT. Antibody binding was detected after sections were incubated in avidin-biotin solution for 45 min (Vector Laboratories). To develop the staining, SG Blue kit (Vector laboratories, Burlingame, CA, USA) was used as the chromogenic substrate for horseradish peroxidase. After immunolabeling, sections were counterstained with nuclear fast red (Sigma-Aldrich, Steinheim, Germany). Coverslipping was done with Pertex mounting medium (Pertex, Medite, Burgdorf, Germany) on dehydrated sections using an ascending ethanol series (50 to 100%) and xylene. Bright-field imaging was done using a Zeiss Axioplan 2 microscope (Carl Zeiss, MicroImaging GmbH, Jena, Germany).

### Pathology grading of p-αS-positive inclusions

Brain pathology was quantified in a set of every 12th serial, sagittal sections of one hemisphere by assessing both perikaryal and neuritic p-αS-labeling. The brain regions of each section were analyzed and the rater determined a mean pathological severity. A semi-quantitative severity score was used in a four-graded scale: Absent (−), mild (+), moderate (++), and severe (+++) p-αS-positive pathology. The person who performed the analysis was blinded towards the mouse genotypes.

### Immunofluorescence, Thioflavin S and pFTAA staining

Brain sections were washed with PBS (3 × 10 min) and mounted on super frost slides. Sections were allowed to air dry for 2 h at room temperature (RT). Mounted and air-dried brain sections were subjected to antigen retrieval by boiling in 10 mM citrate buffer (1.8 mM citric acid, 8.2 mM trisodium citrate, pH 6.0) at 90 °C for 35 min for p-αS, or 80% formic acid for 1 min at RT for epitope specific α-synuclein antibodies, and treated with 5% normal goat or donkey serum in 0.3% Triton-X100 in TBS for 1 h at RT to block unspecific binding. Sections were incubated with primary antibodies (p-αS 1:750, Abcam EP1536Y, Cambridge, United Kingdom; Iba1 1:500, ThermoFisherScientific, Waltham, MA, USA; NeuN 1:500, Millipore, Darmstadt, Germany; αS 34–45 1:200, αS 80–96 1:100, αS 117–122 1:100, BioLegend, San Diego, CA, USA) overnight at 4 °C. The following day, Alexa Fluor 488, 568, or 633 conjugated secondary antibodies (Invitrogen, Waltham, MA, USA, 1:250) were added and incubated for 2 h at RT. Subsequently, labeling with pentamer formyl thiophene acetic acid (pFTAA; stock solution of 1.5 mM in de-ionized water, diluted to a final concentration of 3 μM in PBS) was performed as previously described [29]. Sections were treated for autofluorescence with TrueBlack Lipofuscin Autofluorescence Quencher (Biotium, Fremont, CA, USA) for 30 s at RT. For Thioflavin S (ThioS, Sigma-Aldrich, Steinheim, Germany) staining, sections were incubated for 8 min with 1% w/v ThioS in ddH_2_O. ThioS-stained sections were washed 2 x in 70% EtOH for 3 min and rinsed with ddH_2_O. After air-drying, the sections were coverslipped with Dako Fluorescence mounting medium (Biozol Diagnostika, Cat# S3023). Images were captured on a Zeiss LSM 880 (Zeiss, Oberkochen, Germany) confocal microscope equipped with a spectral scanner.

### Spectral analysis of pFTAA staining

Emission spectra were acquired from 470 to 695 nm and normalized to their respective maxima [30, 31]. Spectra were collected from selected neuronal or microglial cytoplasmic pFTAA-positive inclusions within the brainstem. The ratio of the intensity of emitted light at the red-shifted (584 nm) versus the green-shifted (513 nm) portion was used as a parameter for spectral distinction of different inclusions. These two wavelengths were selected because differences in pFTAA emission were most pronounced for different αS aggregates. Both for neuronal and microglial inclusions, at least three different ROIs per image were calculated. For each mouse, all ROIs from three images were averaged and the mean was taken for statistical analysis (*n* = number of mice; 5–8 mice were analyzed per TG mouse line).

### Immunoassay for αS measurements in brain homogenates

Concentrations of total (human and mouse) αS were determined by a colorimetric HRP-linked immunoassay using the SensoLyte™ Anti-Alpha-Synuclein Quantitative ELISA Kit (AnaSpec, 55550, Fremont, CA, USA). Measurement was conducted according to the manufacturer’s instructions. In brief, formic acid-soluble half-brains were used at 1:2000 (for non-tg mice) or 1:10000 (for tg mice) in dilution buffer (Component C, AnaSpec), added to 96-well plates and co-incubated with detection antibody (1 μg/ml) overnight at 4 °C. After washing, tetramethylbenzidine substrate solution was added and incubated at room temperature until the color was clearly observable. Stop solution was added to block the reaction and absorbance was read promptly on a Mithras LB 940 plate reader (Berthold Technologies, Bad Wildbad, Germany).

### Statistics and image analysis

Statistical analysis was performed using GraphPad Prism 6.0 (GraphPad Software, San Diego, CA, USA). Statistical significance was assessed using ANOVA followed by Bonferroni’s post-hoc test. Data were expressed as indicated in the figure legends. For survival analysis, log-rank test was used. Multiple comparisons of Kaplan-Meier curves were performed with Bonferroni correction. Survival curves were expressed as median incubation times (days). There was no difference between males and females in all the statistical analysis carried out, thus males and females were combined. The grading of p-αS pathology was performed using a semi-quantitative scale ranging from absent (−), mild (+), moderate (++), to severe (+++) by analysis of various brain regions of spontaneously ill TG mice (*n* = 3–5). Percentage of pFTAA-positive inclusions was quantified using in-house written ImageJ macro. Three randomly selected animals from each line were used for the analysis. Three images were analyzed per animal. For spectra and ratios, data were collected from randomly selected *n* = 8 for Prnp-h[A53T]αS, *n* = 8 for Thy1-h[A53T]αS, *n* = 8 for Thy1-h[A30P]αS, and *n* = 5 for Thy1-mαS) mice. Statistical significance was assessed using two-way ANOVA.

## Results

### Symptom onset, life span, and lesions among αS TG mouse lines

**Fig. 1:**
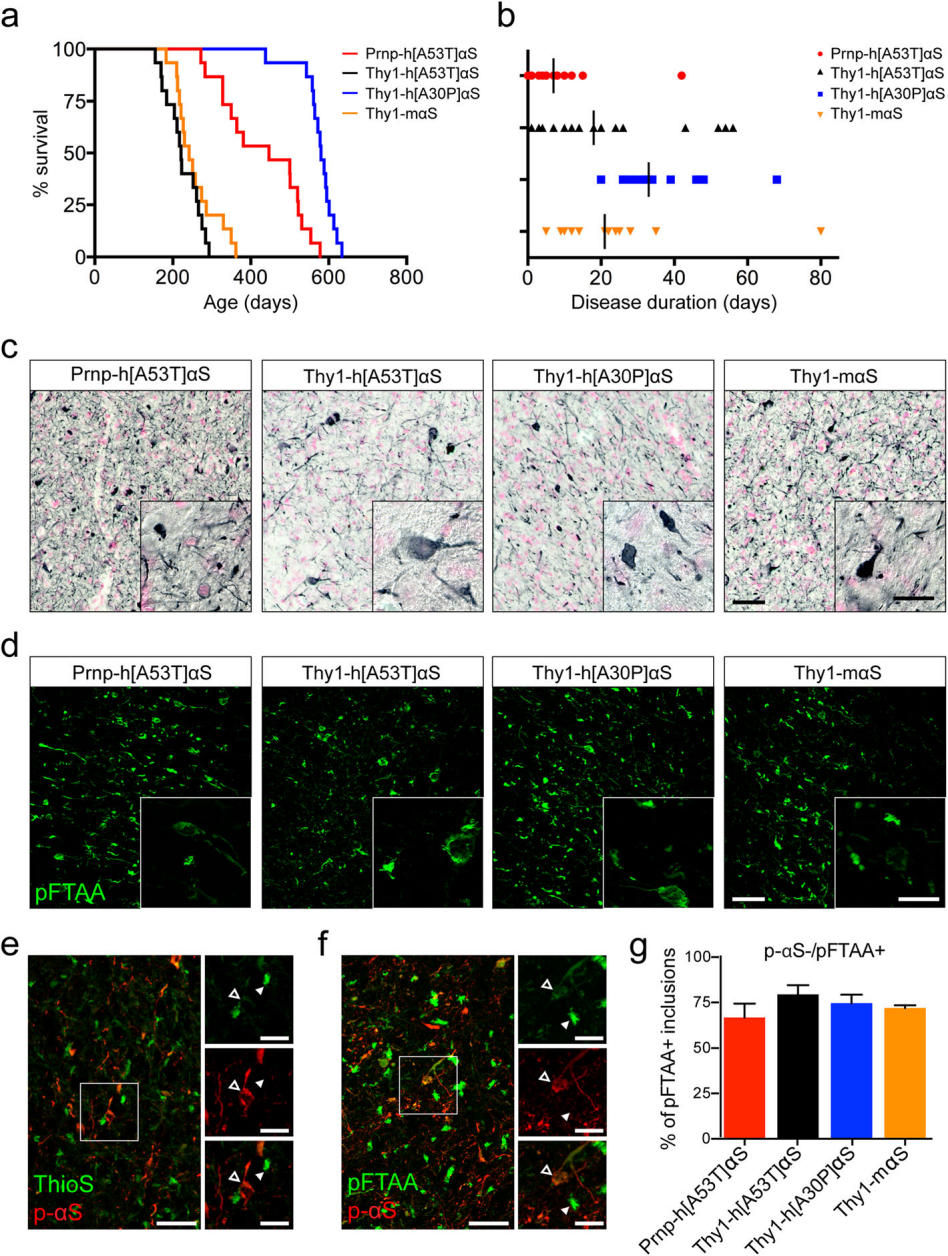
Life span, disease duration, and end-stage αS lesions of different αS TG mouse lines. **(a)** Kaplan-Meier curves for the appearance of clinical end-stage motor signs in Prnp-h[A53T]αS (red curve, median 447 days, *n* = 15), Thy1-h[A53T]αS (black curve, median 221 days, *n* = 15), Thy1-h[A30P]αS (blue curve, median 580 days, *n* = 15), and Thy1-mαS (orange curve, median 242 days, *n* = 15). When survival times of TG lines were compared to each other pair-wise, statistically significant differences were found (Log-rank test, *p* < 0.0001) except for Thy1-h[A53T]αS vs. Thy1-mαS. **(b)** Disease duration starting from onset of motor signs until end-stage phenotype in Prnp-h[A53T]αS (red, median 7 days), Thy1-h[A53T]αS (black, median 18 days), Thy1-h[A30P]αS (blue, median 33 days), and Thy1-mαS (orange, median 21 days). When disease durations of TG lines were compared to each other pair-wise, only Prnp-h[A53T]αS and Thy1-h[30P]αS lines had a statistical difference in their disease duration (One-way ANOVA, Bonferroni’s multiple comparison test, *p* < 0.0001). **(c)** Immunostaining of inclusions labeled with the p-αS antibody, which recognizes phosphorylated αS at serine 129, in Prnp-h[A53T]αS, Thy1-h[A53T]αS, Thy1-h[A30P]αS, and Thy1-mαS mice. Nuclear fast red was used as counterstain. Representative sagittal sections of the midbrain from 12-, 7.3-, 20.8-, and 8.3-month-old mice, respectively, are shown. Scale bars, 50 μm and 20 μm (insert). **(d)** Representative images of pFTAA-positive inclusions in the brainstem of terminally ill Prnp-h[A53T]αS, Thy1-h[A53T]αS, Thy1-h[A30P]αS, and Thy1-mαS mice. Scale bars, 50 μm and 20 μm (insert). **(e)** Fluorescence double-staining for p-αS (red) and ThioS (green) of brainstem pathology in Thy1-h[A30P]αS. Examples of p-αS-positive inclusion (arrowhead outlines) and ThioS-positive aggregate (white arrowheads) are shown in high magnification (inserts). **(f)** Fluorescence double-staining for p-αS (red) and pFTAA (green) of brainstem pathology in Thy1-h[A30P]αS. Note that many of the pFTAA-positive inclusions are not co-labeled with the p-αS antibody. Examples of a p-αS/pFTAA-double-positive inclusion (arrowhead outline) and a pFTAA-positive deposit in absence of p-αS signal (white arrowhead) are in high magnification (inserts). **(g)** Percentage of pFTAA-positive inclusions that are lacking p-αS signal. A similar proportion of non-overlapping pFTAA signal among the lines was found (*n* = 3 mice per mouse line). Results are expressed as mean ± SEM

Disease characteristics (i.e., life span and disease duration) were recorded in Prnp-h[A53T]αS, Thy1-h[A53T]αS, Thy1-h[A30P]αS and Thy1-mαS mice. To this end, 15 animals per mouse line were aged and sacrificed when they displayed the characteristic end-stage neurological signs, i.e. progressive gait instability and/or partial paralysis of the hind limbs (see methods). Results revealed that the Thy1-h[A30P]αS mice are the most long-lived with a median life span of 580 days, followed by Prnp-h[A53T]αS mice (447 days) (Fig. 1a). By contrast, Thy1-h[A53T]αS and Thy1-mαS mice all showed symptoms at an earlier age (221 and 242 days, respectively) (Fig. 1a). The time interval between occurrence of first disease symptoms (slight disturbance in balance, jerky movements) and symptom-related time of sacrifice was defined as the disease duration (Fig. 1b). Of note, the shortest symptomatic phase was observed in Prnp-h[A53T]αS mice and lasted only 7 days.

Brain pathology of the four αS TG mouse lines was examined after the mice were sacrificed. All four lines revealed neuronal p-αS-positive inclusions in both cell soma and neurites. The lesions were most prominent in the brainstem (Fig. 1c). Other regions, including zona incerta, deep cerebellar nuclei showed moderate amounts of αS inclusions, while only sparse αS inclusions were found in the frontal cortex (Supplementary Fig. 1, Supplementary Table 1). Of note, the hippocampus was devoid of any αS pathology in all four lines (Supplementary Fig. 1, Supplementary Table 1). Overall, end-stage αS lesions did not reveal major differences in morphological appearance and regional distribution between the mouse lines (despite some differences in αS levels, Supplementary Fig. 2).

Luminescent conjugated oligothiophenes (LCOs) are dyes that bind to cross-β-sheet structures. These dyes have a flexible backbone that allows changes in their spectral properties depending on the amyloid conformation [32–34]. Recently, pFTAA has been used to detect aggregated αS species in vitro and in vivo [35–37]*.* pFTAA-staining was performed in all four TG lines and pFTAA-positive inclusions were found most robustly in the brainstem, as was the case for p-αS staining (Fig. 1d) but also in all other brain regions with p-αS-positive inclusions. However, double-staining for p-αS and pFTAA was only partially overlapping, and in absence of the p-αS signal pFTAA-positive inclusions appeared much brighter than inclusions that were also p-αS-positive (Fig. 1f). Similar results were observed when stained for p-αS and ThioS (Fig. 1e). Moreover, pFTAA-positive/p-αS-negative inclusions appeared as a compact “ball of threads” and are henceforward referred to as “wool-like inclusions” (Fig. 1f, inserts). In all four lines between 70 and 75% of pFTAA-positive inclusions were p-αS negative (Fig. 1g). No pFTAA-positive staining was found in aged C57BL/6 J wild type (WT) mice (Supplementary Fig. 3).

### pFTAA-positive inclusions are found in microglia and are distinct from neuronal αS aggregates

**Fig. 2:**
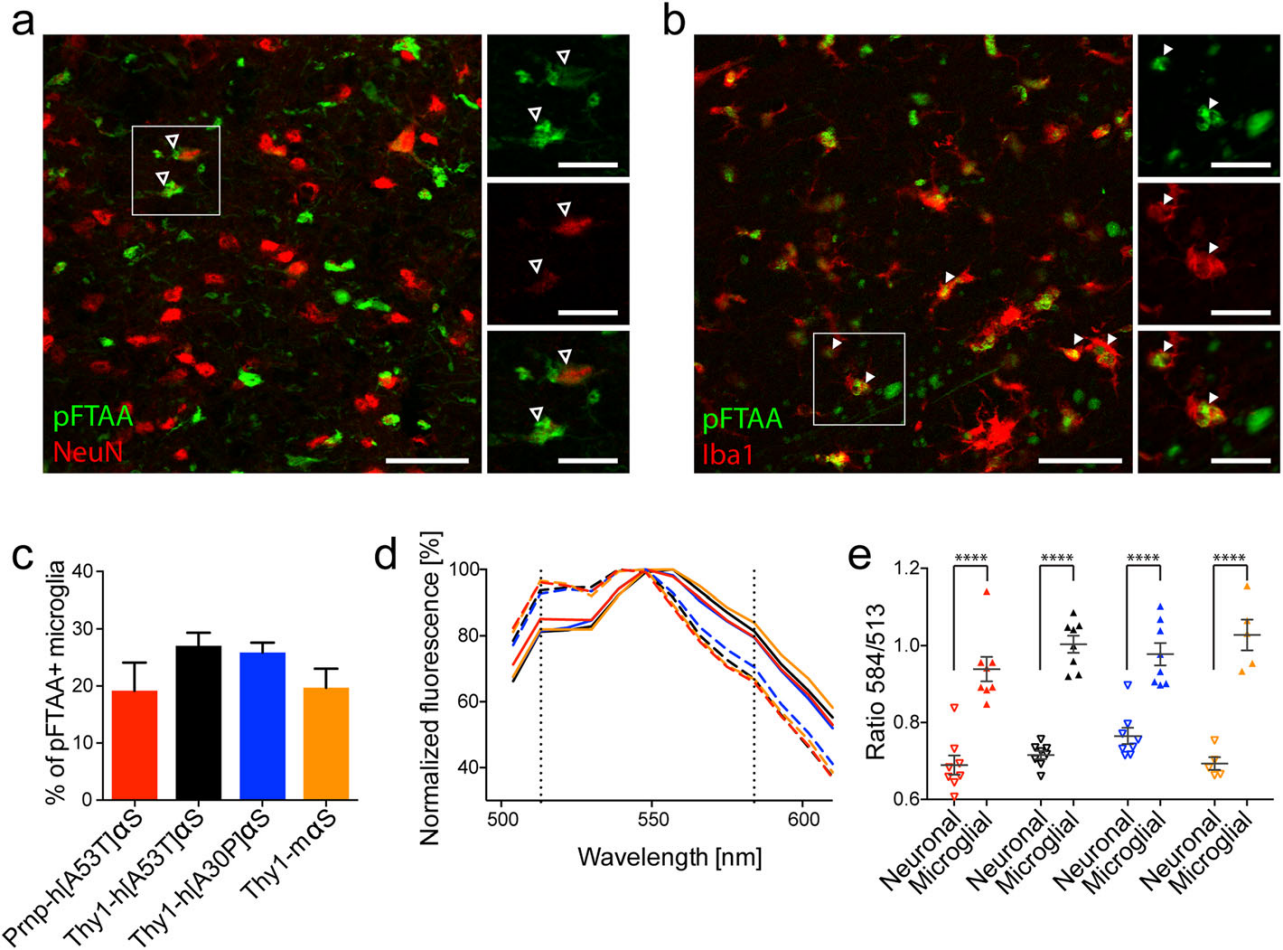
Distinct pFTAA-positive inclusions in neurons and microglia of symptomatic αS TG mice. **(a)** Fluorescence double-staining for pFTAA (green) and NeuN (red) of brainstem pathology in Thy1-h[A30P]αS. Examples of neuronal pFTAA-positive inclusions around the nuclei are shown (arrowhead outlines). Scale bars, 50 μm and 20 μm (inserts). **(b)** Fluorescence double-staining for pFTAA (green) and Iba1 (red) of brainstem pathology in Thy1-h[A30P]αS. Examples of microglial pFTAA-positive inclusions are shown (arrowheads). Scale bars, 50 μm and 20 μm (inserts). **(c)** The percentage of microglia containing pFTAA-positive inclusions for all mouse lines. The proportion of pFTAA-/Iba1-double-positive cells was not significantly different between the lines (*n* = 3 mice per line). Results are expressed as mean ± SEM. (**d**, **e**) Spectral analysis of pFTAA-positive inclusions in neurons and microglia. **(d)** Mean emission spectra of NeuN-positive (dotted lines) and Iba1-positive (solid lines) deposits in Prnp-h[A53T]αS (red, *n* = 8), Thy1-h[A53T]αS (black, *n* = 8), Thy1-h[A30P]αS (blue, *n* = 8), and Thy1-mαS (orange, *n* = 5). Vertical black dotted lines represent the first peak and the shoulder of pFTAA spectra at wavelengths of 513 and 584 nm, respectively. **(e)** The ratio of emission intensity at wavelengths 513 and 584 nm calculated to show the spectral shift of pFTAA upon binding to neuronal (empty triangles) and microglial (solid triangles) inclusions in Prnp-h[A53T]αS (red, *n* = 8), Thy1-h[A53T]αS (black, *n* = 8), Thy1-h[A30P]αS (blue, *n* = 8), and Thy1-mαS (orange, *n* = 5). Two-way ANOVA (cell type x mouse line) revealed a significant effect for cell type [F(1,50) = 218, ****P < 0.0001], but not for mouse line [F(3,50) = 2.156, *P* = 0.1049] or interaction between cell type and mouse line [F(3,50) = 1.842, *P* = 0.1516]. The results are expressed as mean ± SEM

To study the cellular association of the pFTAA-positive inclusions, co-staining for pFTAA and for either the neuronal marker NeuN, the microglia marker Iba1 (Fig. 2), or the astrocytic marker GFAP (data not shown) was performed. As expected, pFTAA-signal that co-localized with NeuN-positive cells appeared morphologically similar to the p-αS staining (Fig. 2a). The pFTAA-positive inclusions that co-localized with Iba1 again had a bright wool-like appearance (Fig. 2b). Some pFTAA-positive structures were observed in astrocytes however by far less abundant than in neurons or microglia (data not shown). Quantification revealed that approximately 25% of total microglia contained pFTAA-positive inclusions and this was similar for all four TG lines (Fig. 2c).

To study conformational differences of inclusions between cell types, spectral analysis (pFTAA/NeuN vs pFTAA/Iba1) was performed (Fig. 2d, e). Spectra were obtained from perikaryal neuronal or microglial inclusions. For all TG mouse lines, there were robust spectral differences between the aggregates in microglia and neurons. However, there was no difference in the spectral signature of the neuronal or microglial inclusions between the lines. These results indicate that microglial inclusions are part of the pathophysiology observed in TG mice and that they are conformationally distinct from neuronal aggregates.

### Microglial inclusions comprise C-terminally truncated αS

**Fig. 3:**
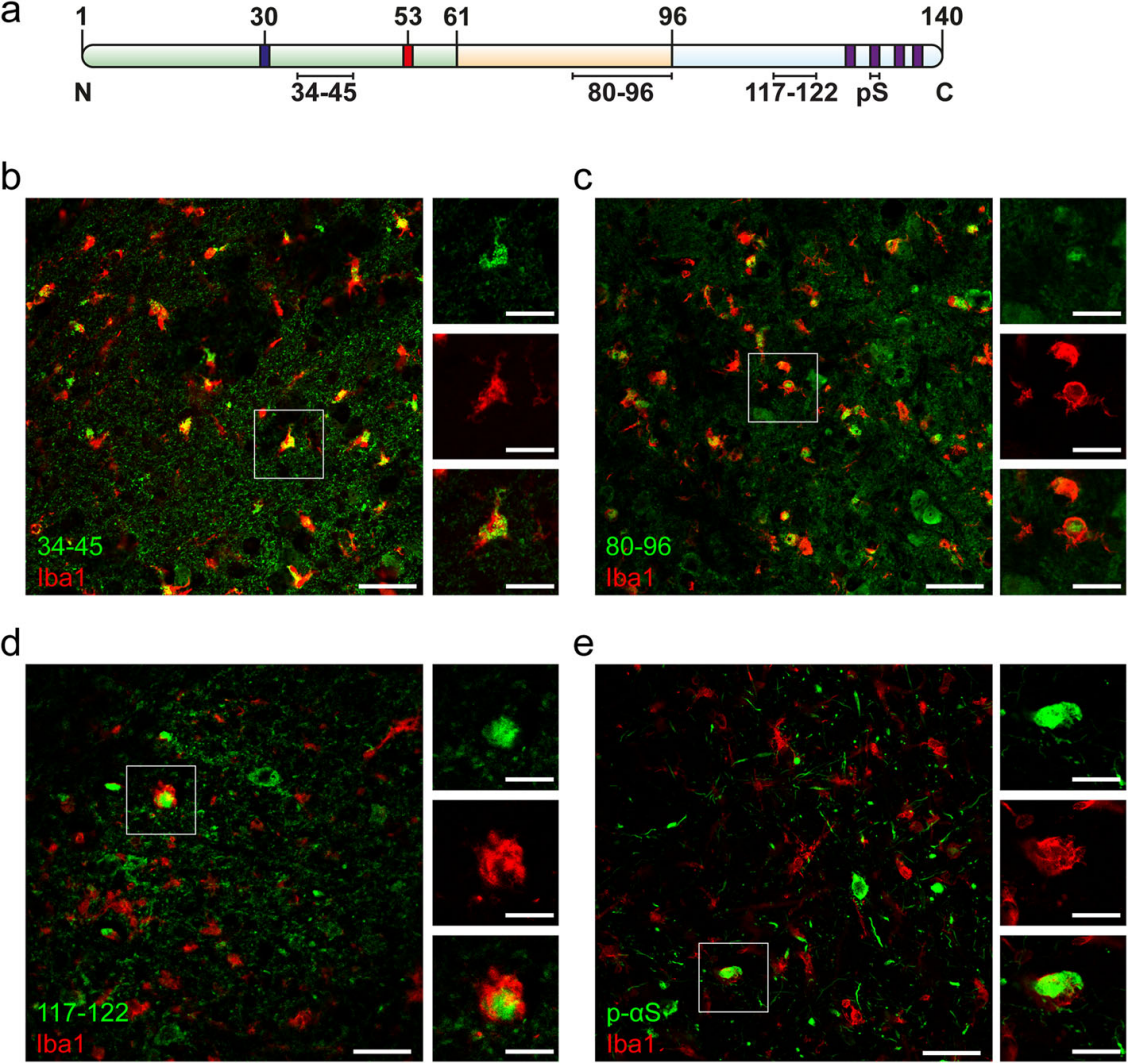
Characterization of microglial αS inclusions in symptomatic αS TG mice. **(a)** Schematic of αS showing the N-terminal region (light green) with PD-linked mutations A30P (blue) and A53T (red), NAC domain (light orange) and C-terminus (light blue) with four phosphorylation sites (purple). Black lines indicate the epitopes of antibodies specific for the N-terminus (34–45), NAC domain (80–96), C-terminus (177–122), and p-αS (phosphorylated αS at serine 129) that were used for immunofluorescence staining. **(b-e)** Co-immunofluorescence staining for Iba1 (red) and epitope-specific αS antibodies (green) in the brainstem of terminally ill Thy1-h[A30P]αS. (**b**) Section of Iba1-positive microglia with 34–45-positive αS aggregates. Note that most Iba1-positive cells are also labeled with anti-αS antibody. Complete overlapping signal of Iba1 and anti-αS 34–45 antibodies in the enlarged images. (**c**) Section of Iba1-positive microglia with 80–96-positive αS inclusions. Note that most Iba1-positive cells are co-localized with anti-αS antibody. Complete overlapping signal of Iba1 and anti-αS 80–96 antibodies in the enlarged images. (**d**) Section of Iba1-positive microglia and 117–122 anti-αS antibody. Note that most Iba1-positive cells are devoid of 117–122 anti-αS antibody staining. A rare occasion with partial overlap of Iba1 and 117–122 anti-αS signal is demonstrated in the enlarged images suggesting microglia phagocytosing αS-positive structure. (**e**) Section of Iba1-positive cells labeled with p-αS antibody. Note that most of the Iba1-positive cells are p-αS-negative. Similar to (d), a rare occasion with partial overlap of Iba1 and anti-p-αS signal is demonstrated in the enlarged images suggesting microglia phagocytosing αS-positive structure. Scale bars, 50 μm and 20 μm (enlarged images)

To assess whether the pFTAA-positive inclusions found in microglia contain αS, double-labeling of the microglial marker Iba1 together with a panel of commercially available anti-αS antibodies specific for both termini and the non-amyloid component (NAC) domain was performed (Fig. 3). Antibodies specific for the N-terminus (epitope between amino acids 34–45) and the NAC region (amino acids 80–96) abundantly co-localized with Iba1-positive microglia, whereas antibodies specific for the C-terminus (amino acids 117–122) and p-αS did not co-localize (Fig. 3). These observations suggest that microglial inclusions contain C-terminally truncated αS. Notably, on some occasions, αS C-terminal-positive structures were found to be associated with Iba1-positive microglia. However, in these instances, these inclusions had a different appearance that was reminiscent of microglial engulfment of αS inclusion-positive structures (Fig. 3d and e, enlarged images).

### Microglial inclusions are already present in presymptomatic TG mice

**Fig. 4:**
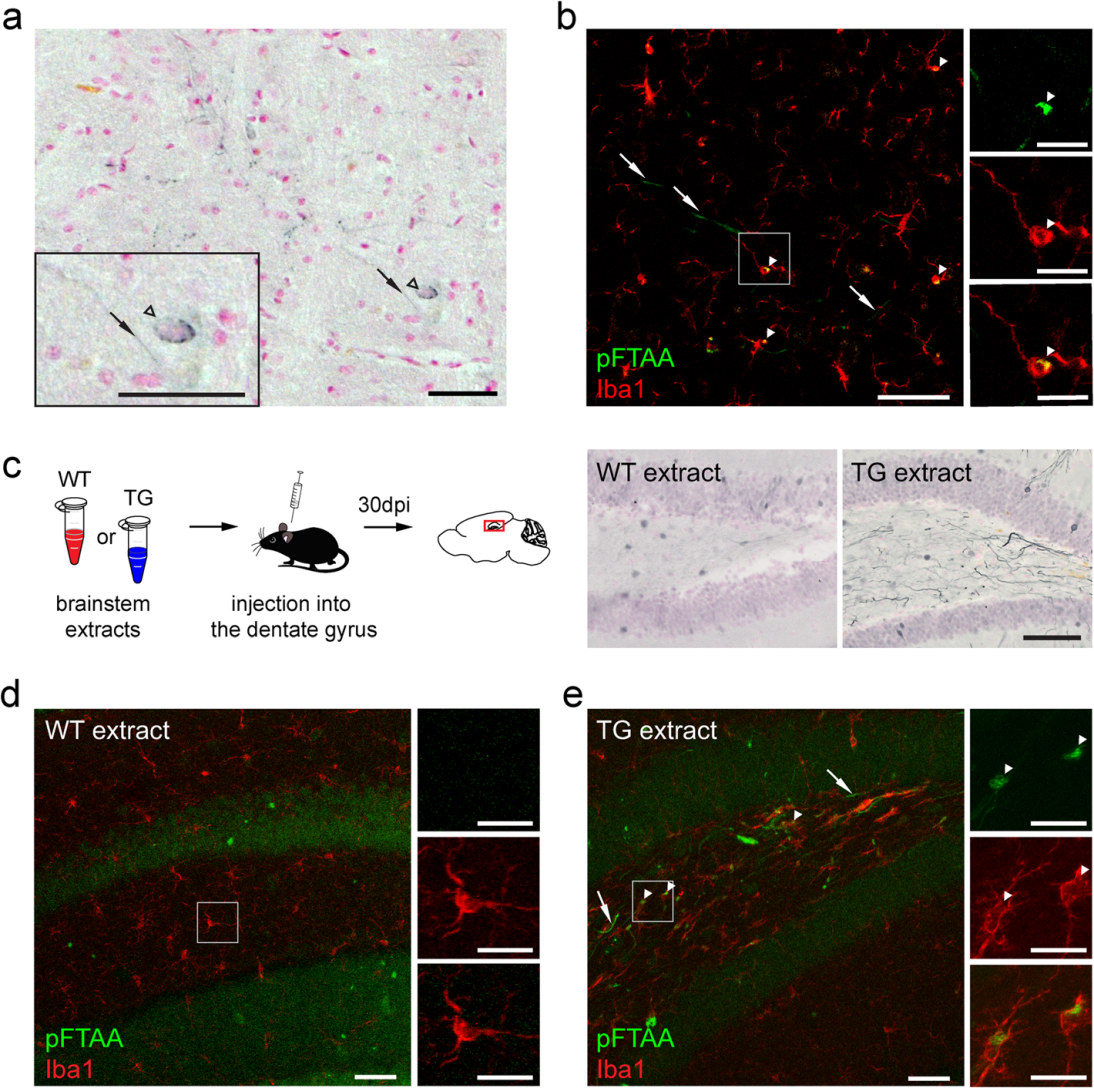
pFTAA-positive microglia in presymptomatic αS TG mice and seeded αS induction model. **(a, b)** Representative brainstem sections of a 15-month-old presymptomatic Thy1-h[A30P]αS mouse, presumed 4–6 weeks before the first symptoms occur. (**a**) Immunostaining of p-αS-positive aggregates (black). Perikaryal (arrowhead outlines) and neuritic (arrows) inclusions are highlighted. Section was counterstained using nuclear fast red. Scale bars, 50 μm and 20 μm (insert). **(b)** Fluorescence double staining of Iba1-positive microglia (red) and pFTAA-positive inclusions (green), showing neuritic pFTAA-positive inclusions (arrows) and pFTAA-positive aggregates in Iba1-positive cells (arrowheads). Example of a pFTAA-positive microglia is shown in high magnification (inserts). Scale bars, 50 μm and 20 μm (inserts). **(c)** Schematic illustration of the intracerebral injection paradigm (left) and sagittal sections of dentate gyrus (DG, site of injection) stained with p-αS antibody (blue) from Thy1-h[A30P]αS mice that have been injected 30 days prior with either brainstem extract from end-stage TG mice (TG extract) or brain extract from WT mice (WT extract). In TG extract-injected mice, abundant p-αS-positive aggregates were detected. In contrast, WT extract did not induce any inclusion. Sections were counterstained using nuclear fast red. Scale bar, 100 μm. **(d, e)** Representative sagittal sections from either WT extract- (**d**) or TG extract-injected (**e**) Thy1-h[A30P]αS mice stained with pFTAA (green) and Iba1 (red). (**d**) No pFTAA-positive inclusions can be found in microglia of WT extract-injected mice. Scale bars, 50 μm and 20 μm (enlarged images). **(e)** In contrast, pFTAA-positive inclusions are found in Iba1-positive microglia in DG from Thy1-h[A30P]αS mice. Examples of neuritic pFTAA-positive inclusions (arrows) and pFTAA-positive deposits in microglia (arrowheads) are highlighted. Scale bars, 50 μm and 20 μm (enlarged images)

To investigate whether αS inclusions in microglia are a feature of end-stage pathology or if they develop alongside neuronal αS lesions before the first motor signs occur, mice were analysed at presymptomatic stage (Fig. 4a, b). Due to its extended survival time, the Thy1-h[A30P]αS mouse line was initially chosen for this analysis (Fig. 1a). Intriguingly, at the time of the first p-αS-positive neuronal inclusions of around 15 months of age there were also scarce pFTAA-positive microglial inclusions present (Fig. 4a, b). Presymptomatic Thy1-h[A53T]αS also showed similar co-occurrence (Supplementary Fig. 4). This indicates that neuronal and microglial inclusions may develop around the same time, which further highlights a potential role of microglia in pathology.

### Seeded induction of neuronal αS inclusions is also accompanied by microglial inclusions

To further study the link between neuronal and microglial αS inclusions, neuronal αS pathology was induced in young, presymptomatic Thy1-h[A30P]αS mice by seeding [28]. To this end, brainstem extract from end-stage Thy1-h[A30P]αS TG mice or brain extract from WT mice were injected into the hippocampus of 2–4-month-old Thy1-h[A30P]αS mice (Fig. 4c-e). As expected, 30 days post-injection, mice inoculated with TG brainstem homogenate revealed p-αS-positive neuronal inclusions around the injection site (Fig. 4c). In addition, pFTAA-positive microglial inclusions were also present in vicinity of the injection site (Fig. 4e). In contrast, mice injected with control WT brain homogenate did not develop any neuronal or microglial lesions (Fig. 4d).

## Discussion

The initial aim of the present work was to study disease progression and features of αS lesions among TG mouse models of α-synucleinopathies and their correlation with αS conformers. The mouse lines revealed major differences in age-of-symptom onset and disease progression. Postmortem analysis though revealed an overall very similar appearance and distribution of the αS lesions in all the lines. However, strikingly, in addition to neuronal lesions, we found αS-positive inclusions in microglia in all four lines. Although it had not been reported with such an abundance before, previous studies made note of apparent αS aggregates in microglia in viral vector-based and TG αS-overexpressing mouse models after seeding [38–41].

This unexpected finding of robust inclusions in microglia in αS TG mice was initially made through the analysis with the amyloid-binding dye pFTAA and was subsequently confirmed through ThioS-positive labeling. LCOs have previously been reported to bind and discriminate structural variants of PrP [32], Aβ [33, 42], and tau aggregates [29, 43]. More recently, studies also showed that αS aggregates can be detected in solution and in an in vitro seeding assay using pFTAA [35, 37] and in disease samples using other LCOs [7, 8, 44]. Although we did not succeed to distinguish A53T from A30P αS aggregates readily with pFTAA, we found that spectral analysis using pFTAA could clearly distinguish the neuronal from the microglial inclusions suggesting conformational differences of the inclusions between these cell types.

Differences in structural features between neuronal and microglial inclusions are also in line with stainings using different αS antibodies. While N-terminal- and NAC domain-specific αS antibodies also labeled microglial inclusions, the C-terminal-specific antibody did not detect microglial inclusions (including p-αS at serine 129). Therefore, microglial inclusions are easily overlooked when using p-αS antibodies. Notably, in rare cases, we also observed microglia-associated inclusions that were p-αS-positive. However, in most such instances, inclusions appeared to be part of a neuronal element that appeared to be engulfed actively by a microglial cell [39]. This observation is reminiscent of microglial phagocytosis of neurons filled with tau filaments [45].

Our data suggest that there is a link between the neuronal and microglial inclusions in αS TG mice, since they always co-occur and we never observed only neuronal or only microglial αS inclusions. Also, at early presymptomatic stages or upon seeded induction of αS inclusions, neuronal inclusions were always accompanied by microglial inclusions in close vicinity of the neuronal inclusions. It is possible that microglial inclusions are the results of phagocytosed neuronal elements or uptake of neuron-released αS [39, 41] with subsequent removal of the C-terminus, which leads to structural rearrangement and changes in pFTAA emission spectra. It is also conceivable that microglia take up neuronally-secreted soluble oligomeric αS species [46], which then assemble within the microglia to filamentous αS aggregates. It is known that different cellular environments influence the composition and conformation of proteopathic seeds [47], exemplified by αS aggregates in oligodendrocytes that are more compact and reveal higher seeding potency than their neuronal counterparts [6]. Finally, αS is expressed at low levels in microglia under homeostatic conditions [48]. It is therefore plausible that activated microglia upregulate αS expression and that microglial inclusions are partly formed by aggregation of microglia-generated αS.

An abundance of microglial αS inclusions described here has not been reported in humans [1], albeit very recently, microglia αS inclusions in the human olfactory bulb of PD patients have been described [49]. Furthermore, seeding-prone αS species were detected in human microglial exosomes isolated from CSF of sporadic PD and MSA patients [50] and microglia are involved in the spreading of αS lesions [51, 52]. These studies raise the possibility that αS aggregates in microglia in α-synucleinopathies are more common than previously thought and that they may also contribute to disease progression. If, however, abundant microglial αS inclusions turn out to be restricted to αS overexpressing TG mouse models, this knowledge is important when αS TG models are utilized in preclinical-translation studies.

## Supplementary information

**Additional file 1: Figure S1** Histopathology in αS TG mouse lines. **Figure S2.** Total brain αS levels in TG and WT mice. **Figure S3.** Negative controls for p-αS immunohistochemistry and pFTAA staining. **Figure S4.** p-αS immunohistochemistry and pFTAA staining in presymptomatic Thy1-h[A53T]αS mice. **Table S1.** Grading of p-αS-positive αS pathology in TG mice

## Data Availability

The authors will provide upon request raw data and material (some of them via material transfer agreement).
