# Supplementary material for: Prominent microglial inclusions in transgenic mouse models of α-synucleinopathy that are distinct from neuronal lesions
Source: Acta Neuropathol Commun. 2020 Aug 12;8:133. doi: 10.1186/s40478-020-00993-8 (PMC7425556; doi:10.1186/s40478-020-00993-8)
Supplement: Supplementary file 1 — Additional file 1: Figure S1 Histopathology in αS TG mouse lines. Figure S2. Total brain αS levels in TG and WT mice. Figure S3. Negative controls for p-αS immunohistochemistry and pFTAA staining. Figure S4. p-αS immunohistochemistry and pFTAA staining in presymptomatic Thy1-h[A53T]αS mice. Table S1. Grading of p-αS-positive αS pathology in TG mice [file 40478_2020_993_MOESM1_ESM.docx]

**SUPPLEMENTARY FIGURES AND TABLE**


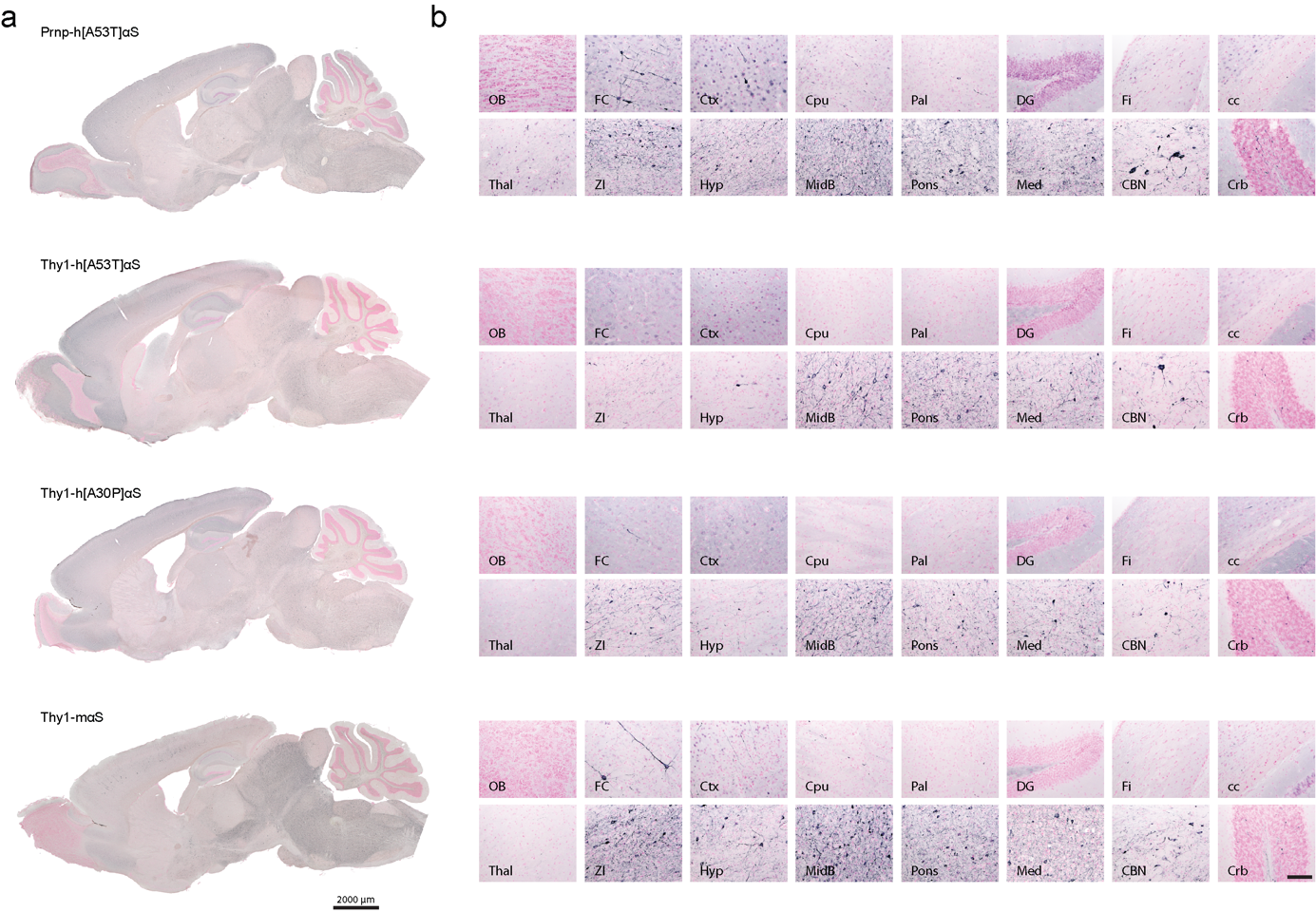


**Fig. S1** Histopathology in αS TG mouse lines. Immunohistochemistry of p-αS-positive αS deposits in terminally ill Prnp-h[A53T]αS, Thy1-h[A53T]αS, Thy1-h[A30P]αS, and Thy1-mαS mice. Sections were counterstained using nuclear fast red. **(a**) Representative sagittal brain sections of Prnp-h[A53T]αS, Thy1-h[A53T]αS, Thy1-h[A30P]αS, and Thy1-mαS mice at 12, 7.3, 20.8, and 8.3 months of age, respectively, are shown. Scale bar, 2000 µm. **(b)** Close-up images of different brain regions from each mouse line in (A) are shown. Scale bar, 100 µm. OB: Olfactory bulb, FC: Frontal cortex, Ctx: Cortex, Cpu: Caudate putamen, Pal: Pallidum, DG: Dentate gyrus, Fi: Fimbria, cc: Corpus callosum, Thal: Thalamus, ZI: Zona incerta, Hyp: Hypothalamus, MidB: Midbrain, Pons: Pons, Med: Medulla, CBN: Cerebellar nuclei, Crb: Cerebellum.


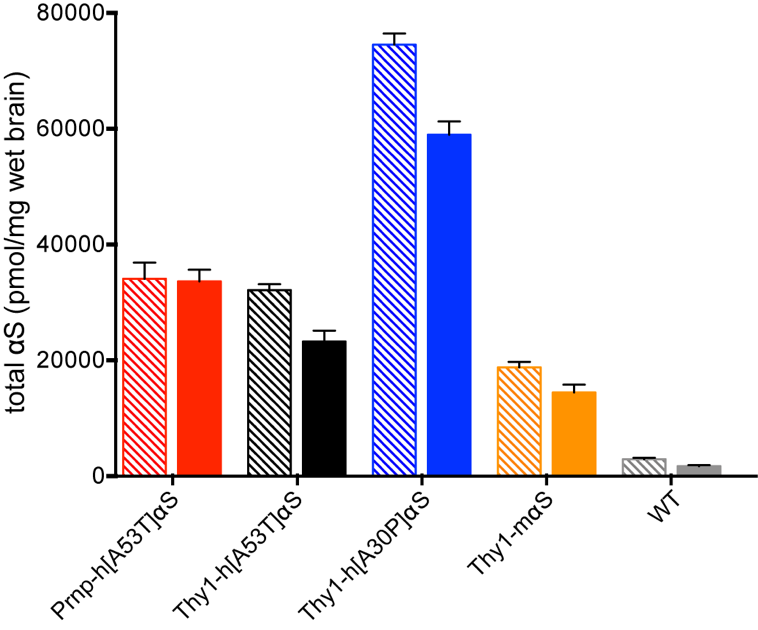


**Fig. S2** Total brain αS levels in TG and WT mice. Levels of αS in formic acid extracted-brain homogenates. Total (mouse and human) αS protein levels in the brains of end-stage (solid bars) TG Prnp-h[A53T]αS (red, n = 4), Thy1-h[A53T]αS (black, n = 4), Thy1-h[A30P]αS (blue, n = 4), and Thy1-mαS (orange, n = 4) mice at 14, 9, 18 and 8 months of mean age (end-stage), respectively, as well as mean age 25-month-old WT (gray, n = 7) mice are shown. Protein levels are also shown for young, 2-month-old mice of all lines (patterned bars, n = 5-8).


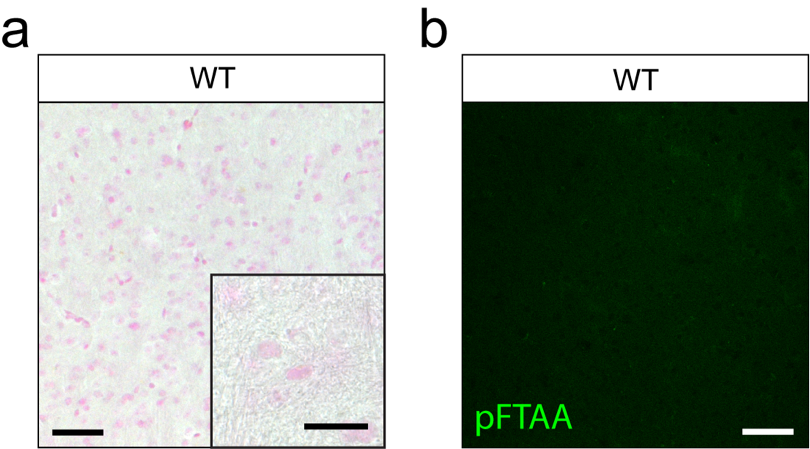


**Fig. S3**  Negative controls for p-αS immunohistochemistry and pFTAA staining. **(a)** Immunostaining of p-αS antibody in the brainstem of a 19.5-month-old C57BL/6J wild-type (WT) mouse. Nuclear fast red was used as counterstain. No p-αS staining was detected. Scale bars, 50 µm and 20 µm (insert). **(b)** pFTAA staining in the brainstem of the same WT mouse. No pFTAA signal was detected. Scale bar, 50 µm.


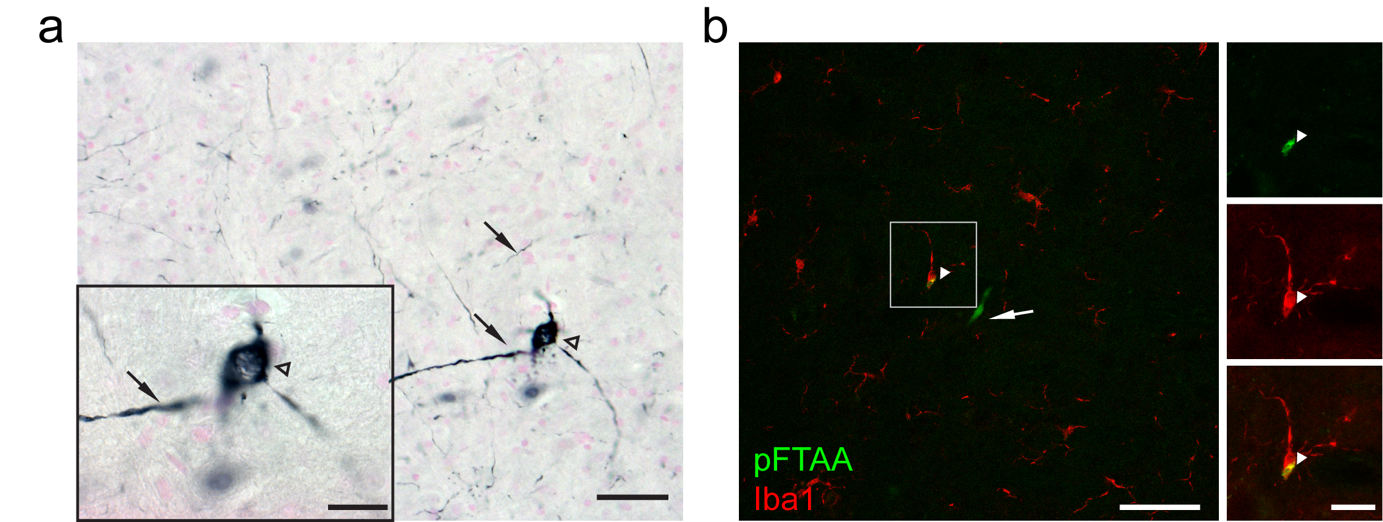


**Fig. S4** p-αS immunohistochemistry and pFTAA staining in presymptomatic Thy1-h[A53T]αS mice. **(a, b)** Representative brainstem sections of a 7-month-old presymptomatic Thy1-h[A53T]αS mouse, presumed 2-3 weeks before the first symptoms occur. (**a**) Immunostained p-αS-positive aggregates (black). Perikaryal (arrowhead outline) and neuritic (arrows) inclusions are highlighted. Section was counterstained using nuclear fast red. Scale bars, 50 µm and 20 µm (insert). **(b)** Fluorescence double labeling of Iba1 (red) and pFTAA (green) reveals an inclusion-positive microglia. Neuritic pFTAA-positive inclusion (arrow) and the pFTAA-positive aggregate in the Iba1-positive cell (arrowhead) are shown. Example of a pFTAA-positive microglia is shown in high magnification (inserts). Scale bars, 50 µm and 20 µm (inserts).

**Table S1 Grading of p-αS-positive αS pathology in TG mice.**

|  | Prnp-h[A53T]αS | Thy1-h[A53T]αS | Thy1-h[A30P]αS | Thy1-mαS |
| --- | --- | --- | --- | --- |
| Olfactory bulb | - | - | - | - |
| Motor cortex | ++ | + | + | ++ |
| Somatosensory areas | + | + | + | + |
| Hippocampus | - | - | - | - |
| Subiculum | - | - | - | - |
| Fimbria | - | - | - | - |
| Corpus callosum | - | - | - | - |
| Internal capsule | ++ | + | ++ | + |
| Striatum | + | - | + | + |
| Pallidum | ++ | + | + | ++ |
| Amygdala | - | - | - | - |
| Thalamus | + | - | - | - |
| Zona incerta | +++ | + | ++ | +++ |
| Superior colliculus | ++ | +++ | ++ | +++ |
| Inferior colliculus | + | + | + | + |
| Periaqueductal grey | +++ | +++ | +++ | +++ |
| Midbrain reticular nucleus | +++ | +++ | +++ | +++ |
| Substantia nigra | - | - | - | - |
| Pons | +++ | +++ | +++ | +++ |
| Medulla | +++ | +++ | +++ | +++ |
| Cerebellar cortex | ++ | + | + | ++ |
| Cerebellar nuclei | ++ | ++ | ++ | ++ |

Analysis of various brain regions of spontaneously ill TG mice (n = 3-5). Grading of p-αS-positive αS pathology was performed using a semi-quantitative scale ranging from absent (-), mild (+), moderate (++) to severe (+++).
